# Siderophore-Mediated Interactions Determine the Disease Suppressiveness of Microbial Consortia

**DOI:** 10.1128/mSystems.00811-19

**Published:** 2020-06-30

**Authors:** Shaohua Gu, Tianjie Yang, Zhengying Shao, Tao Wang, Kehao Cao, Alexandre Jousset, Ville-Petri Friman, Cyrus Mallon, Xinlan Mei, Zhong Wei, Yangchun Xu, Qirong Shen, Thomas Pommier

**Affiliations:** a Jiangsu Provincial Key Lab for Organic Solid Waste Utilization, Key Lab of Plant Immunity, Jiangsu Collaborative Innovation Center for Solid Organic Waste Resource Utilization, National Engineering Research Center for Organic-based Fertilizers, Nanjing Agricultural University, Nanjing, People’s Republic of China; b Department of Biology, University of York, York, United Kingdom; c Groningen Institute for Evolutionary Life Sciences (GELIFES), University of Groningen, Groningen, The Netherlands; d Univ Lyon, Université Claude Bernard Lyon 1, CNRS, INRAE, VetAgro Sup, UMR Ecologie Microbienne, Villeurbanne, France; Rensselaer Polytechnic Institute

**Keywords:** Siderophore, microbial interactions, plant health, plant pathogens, soil microbiology

## Abstract

Interactions between plant pathogens and root-associated microbes play an important role in determining disease outcomes. While several studies have suggested that steering these interactions may improve plant health, such approaches have remained challenging in practice. Because of low iron availability in most soils, competition for iron via secreted siderophore molecules might influence microbial interaction outcomes. Here, we tested if bacterial interactions mediated by iron-scavenging siderophores can be used to predict the disease suppressiveness of microbial consortia against soilborne Ralstonia solanacearum, a bacterial pathogen in the tomato rhizosphere. Iron availability significantly affected the interactions within inoculated consortia and between the consortia and the pathogen. We observed contrasting effects of siderophores and other nonsiderophore metabolites on the pathogen growth, while the siderophore effects were relatively much stronger. Specifically, disease incidence was reduced *in vivo* when the inoculated consortia produced siderophores that the pathogen could not use for its own growth. Employing siderophore-mediated interactions to engineer functionally robust microbial inoculants shows promise in protecting plants from soilborne pathogens.

**IMPORTANCE** Soil-borne pathogens cause high losses in crop yields globally. The development of environmentally friendly approaches is urgently needed, but is often constrained by complex interactions between root-associated microbes and pathogens. Here, we demonstrate that the interactions within microbial consortia mediated by iron-scavenging siderophores play an important role in reducing pathogen infection and enhancing plant health. This study provides a promising and novel research direction for dealing with a wide range of microbial infections through iron exploitation, which is important for the colonization and infection of both plant and human hosts by pathogens.

## Abstract (precis)

Soil-borne pathogens cause high losses in crop yields globally. The development of environmentally friendly approaches is urgently needed, but is often constrained by complex interactions between root-associated microbes and pathogens. Here, we demonstrate that the interactions within microbial consortia mediated by iron-scavenging siderophores play an important role in reducing pathogen infection and enhancing plant health. This study provides a promising and novel research direction for dealing with a wide range of microbial infections through iron exploitation, which is important for the colonization and infection of both plant and human hosts by pathogens.

## INTRODUCTION

Soil-borne pathogens and pests represent a serious threat to agricultural production, causing up to 30% yield losses globally (1). One environmentally sustainable way to improve plant health is to take advantage of plant-associated microorganisms that form the first line of defense against pathogens by preventing their growth and subsequent virulence (2–5). The functionality of these natural communities is often compromised in agricultural soils due to the extinction of several beneficial strains via high-intensity agricultural practices (6, 7). Restoring microbiome ability to efficiently suppress soilborne diseases could, therefore, play a central element for future food security. While several attempts have been made to augment microbiome functioning by introducing potentially beneficial strains or consortia into the soil (8–10), the outcomes are still highly variable. One explanation for this may be our poor understanding of the underlying suppressive and facilitative ecological interactions within the rhizosphere, which could limit the success of microbe-mediated manipulations (11–13). In this study, we focused on understanding the role of competition for iron in engineering disease-suppressive microbial inoculants against the plant-pathogenic bacterium Ralstonia solanacearum.

The diversity of bacterial communities has been acknowledged to play an important role in the resistance to pathogen infections (13, 14) and this positive relationship is often thought to arise as a result of interactions within the bacterial communities (15–17). Specifically, metabolic interactions and resource competition have been shown to be important in determining pathogen infections in the soil (18, 19), and how these interactions take place within the inoculated consortium and between the consortium and the pathogen is often essential for predicting disease outcomes (12). For example, facilitative metabolic interactions within inoculated consortia can potentially promote pathogen growth if the pathogen can also use the exchanged metabolites for growth (12, 20–22). However, if the metabolic interactions are more specific, they might only benefit the members of the inoculated consortium and have no effect, or even negative effects, on pathogen growth via resource competition. Interference competition via direct antagonism has also been shown to be important (23). For example, production of secondary metabolites can drive negative interactions between the inoculated strains, but can also indirectly inhibit the pathogen if it is susceptible to these metabolites (19, 23–25). Alternatively, these secondary metabolites could be directly targeted toward the pathogen without having any negative effect on the inoculated consortium (26). Together, these findings suggest that steering the interactions in microbial communities to suppress soilborne pathogens requires a profound understanding of the underlying ecological interactions both within the inoculated consortium and between the consortium and the pathogen.

One potential way to manipulate microbial interactions could be via the availability of limiting resources, such as carbon, phosphorus, and nitrogen, which are essential for bacterial growth (13, 27, 28). Here, we chose to study the effects of iron as another important limiting resource in mediating microbial competition in the soil. Iron is important for bacterial growth and metabolism (e.g., reduction of oxygen for ATP synthesis and reduction of riboside precursors of DNA) and the demand for iron often exceeds the available iron concentrations in the soil rhizosphere, except for in highly acidic soils, which typically have high ferric solubility (29–31). To capture iron, bacteria either secrete or utilize at least one type of high-affinity iron-chelating compounds called siderophores to compete for iron with other bacterial taxa (30–32). These siderophores can be highly specific and only recognized by the receptors of the siderophore-producing strains, thereby enhancing competition for iron (23, 33). Alternatively, siderophores can be produced as public goods and taken up by other bacteria (30, 34), which could support potential siderophore-mediated facilitation between strains (33, 35, 36). Consequently, the strength and type of interactions within bacterial communities might be determined by iron competition, which could also affect pathogen growth and disease outcomes. Here, we tested if siderophore-mediated interactions between inoculated consortia and the pathogen can be used as an efficient strategy to design suppressive microbial inoculants.

To evaluate the validity of such a strategy, we conducted microcosm experiments where we first designed microbial consortia and measured how they interacted with each other and with the pathogen via siderophores and other secondary (nonsiderophore) metabolites (for more details, see the Materials and Methods section). Furthermore, we used greenhouse experiments to explore how siderophore-mediated interactions within the consortium and between the consortium and the pathogen affected the outcomes of bacterial wilt disease using tomato as a host plant species. In these experiments, we found that siderophores played a key role in shaping the competitive interactions within the consortium and between the consortium and the pathogen, and, in addition, that the strength of these interactions could be used to predict pathogen invasion in the tomato rhizosphere. Competition for iron could thus be used as a framework to design suppressive microbial inoculants that provide an efficient and predictable strategy to control R. solanacearum disease outbreaks.

## RESULTS

### Siderophore production by each member of inoculated consortia.

Five closely related but nonpathogenic *Ralstonia* strains (Ralstonia mannitolilytica QL-A2, Ralstonia mannitolilytica QL-A3, Ralstonia pickettii QL-A6, Ralstonia taiwanensis QL-117, and Ralstonia pickettii QL-140) were used to construct inoculated consortia. These species were chosen as they often cooccur with the pathogenic R. solanacearum strains in China and have been well characterized in our previous studies (13, 27, 28). Siderophore production was indirectly measured using the chrome azurol S (CAS) assay, which can reliably provide a relative comparison between different strains and communities (for more details, see the Materials and Methods section). The mean siderophore production of each inoculated strain grown in monoculture was significantly higher in iron-limited than in iron-rich growth conditions (*P < *0.001) (Fig. S1 in the supplemental material). Compared to the background signal of no siderophore production (7.67 μmol/liter), all inoculated strains showed lower values under iron-rich conditions (Fig. S1). This suggests that few siderophores were produced when iron was readily available. Siderophore production by the different consortium members spanned from 3.06 μmol/liter in iron-rich conditions to 77.95 μmol/liter in iron-limited conditions (Fig. S1). Specifically, the strains QL-A6 and QL-140 produced surprisingly large amounts of siderophores in iron-limited conditions (77.95 and 37.81 μmol/liter, respectively) (Fig. S1).

10.1128/mSystems.00811-19.1 FIG S1 Siderophore production of defined siderophore nonproducers (deletion mutants, left to the vertical line) and inoculated strains in iron-rich (purple, A) and iron-limited (yellow, B) conditions (right to the vertical line), based on a CAS assay. The averaged siderophore production values of the two siderophore-deficient mutants represented a cutoff value (purple and yellow dashed lines) to distinguish background CAS activity from real siderophore production. Data show the mean of four independent experiments and bars indicate the standard deviation. Different lowercase letters above bars represent significances between strains measured in iron-rich (A) and iron-limited (B) conditions at *P* < 0.05 (Duncan’s multiple range test). The differences in siderophore production by the same strain in both iron-rich and iron-limited conditions were determined by paired two-sided Student’s *t* test (*, *P* < 0.05; **, *P* < 0.01; ***, *P* < 0.001; NS represents a nonsignificant difference). Download FIG S1, TIF file, 0.8 MB. Copyright © 2020 Gu et al. 2020 Gu et al. This content is distributed under the terms of the Creative Commons Attribution 4.0 International license.

### Siderophore and nonsiderophore metabolite-mediated effects of inoculated consortia members on pathogen growth.

**FIG 1:**
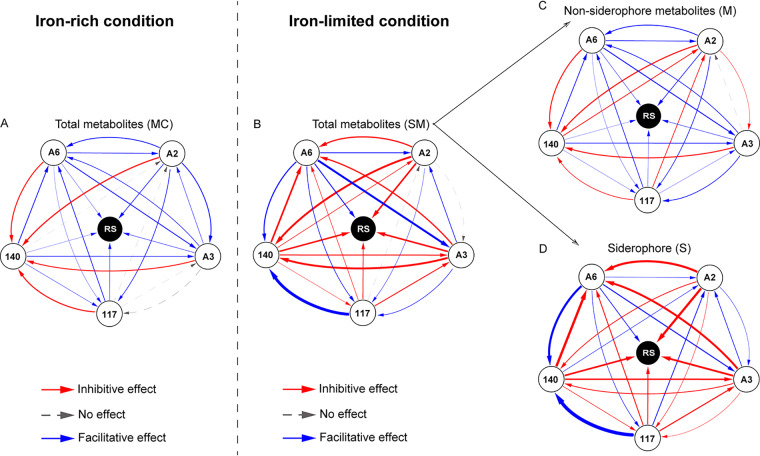
The interactions mediated by siderophores and nonsiderophore metabolites within the inoculated consortia and between the inoculated strains and the pathogen. Network diagram showing the strength and directionality of all pairwise interactions mediated by different metabolites between inoculated strains and the effect of inoculated strains on the growth of the pathogen in iron-rich (A) and iron-limited (B) conditions. The thickness of lines represents the strength of the facilitative or inhibitive relationships and red and blue colors highlight inhibitive and facilitative effects, respectively. Both nonsiderophore metabolite-mediated (C) and siderophore-mediated (D) interactions within the inoculated consortia affected the growth of pathogen in iron-limited conditions. Nonsiderophore metabolite effects cover the effects of all other secondary metabolites, except for siderophores, produced in the iron-limited supernatant. The abbreviations A2, A3, A6, 117, 140, and RS represent the inoculated strains QL-A2, QL-A3, QL-A6, QL-117, QL-140, and the pathogen R. solanacearum, respectively.

To disentangle the effects of siderophores (S) and nonsiderophore metabolites (M) on pathogen growth, we first determined the combined effects of total metabolites (SM) produced by each consortium member under iron-rich and iron-limited conditions (Fig. 1A and B). While the total metabolites of all five consortium members facilitated pathogen growth under iron-rich conditions (Fig. 1A), their effects on pathogen growth varied from inhibitive to facilitative under iron-limited conditions. More specifically, four out of five consortia members (Ralstonia mannitolilytica QL-A2, Ralstonia mannitolilytica QL-A3, Ralstonia taiwanensis QL-117 and Ralstonia pickettii QL-140) inhibited, while Ralstonia pickettii QL-A6 strain promoted the growth of the pathogen (Fig. 1B and Fig. S2).

10.1128/mSystems.00811-19.2 FIG S2 Total metabolite (siderophore and nonsiderophore) effects of inoculated strains on pathogen growth. The direct total metabolite effect on the pathogen growth were measured under iron-rich (MC, purple) and iron-limited (SM, yellow) conditions. The nonsiderophore metabolite effects (M, cyan-blue) on the pathogen growth under iron-limited condition are expressed as the effect by iron-limited supernatant replenished with excess of iron to chelate produced siderophores in iron-limited conditions. To quantify the effects of siderophores only (S, black), we subtracted the nonsiderophore metabolite effect from the total metabolite effect (SM − M = S). Data show the mean of four independent experiments and error bars indicate the standard deviation. Lowercase letters above each bar represent the significance of each strain’s effect under different treatments at *P* < 0.05 (Duncan’s multiple range test). Download FIG S2, TIF file, 2.5 MB. Copyright © 2020 Gu et al. 2020 Gu et al. This content is distributed under the terms of the Creative Commons Attribution 4.0 International license.

To determine the effects of nonsiderophore metabolites (M), iron chelation was implemented into the previous assays to inactivate all siderophores present in the supernatant. Interestingly, this siderophore inactivation turned all inhibitory interactions into facilitative (Fig. 1C and Fig. S2) and these effects showed similar magnitude compared to the metabolite control treatment (MC), where supernatant was derived from iron-rich growth conditions and thus contained very few siderophores (Fig. 1A and C and Fig. S2) (all *P* > 0.05). To estimate the siderophore (S)-mediated effects, we subtracted nonsiderophore metabolite (M) effects from the combined effects (SM). Our results showed that siderophore-mediated effects by each consortium member mainly inhibited the pathogen growth under iron-limited conditions (Fig. 1D and Fig. S2), which was very similar to the combined effects of the total metabolites (SM) in the same growth conditions (Fig. 1B and D and Fig. S2) (three out of five were *P* > 0.05). Altogether these results suggest that siderophore-mediated effects play a key role in triggering pathogen inhibition and that, although these effects depended on the identity of the consortium members, the magnitude of siderophore-mediated effects was always relatively stronger than nonsiderophore metabolite-mediated interactions.

### Siderophore and nonsiderophore metabolite-mediated growth effects between inoculated consortia members.

We applied the same strategy described previously to disentangle siderophore-mediated effects from nonsiderophore metabolite-mediated effects on the growth of each member of the consortium in both iron-rich and iron-limited conditions (Fig. 1A and B). While the total metabolite-mediated effects under iron-rich conditions mostly showed facilitative effects (only 4 out of 20 pairs showed inhibition on strain growth) (Fig. 1A), they inhibited each other’s growth under iron-limited conditions (only 7 out of 20 pairwise were facilitative) (Fig. 1B). As observed with R. solanacearum, four out of five consortium members (Ralstonia mannitolilytica QL-A2, Ralstonia mannitolilytica QL-A3, Ralstonia taiwanensis QL-117, and Ralstonia pickettii QL-140) mediated inhibitive effects in iron-limited conditions, while the R. pickettii QL-A6 strain mediated facilitative effects between all consortium members (Fig. 1B and Fig. S3) (*P* < 0.01). More specifically, R. pickettii QL-140 inhibited all other consortium members; R. mannitolilytica QL-A2 inhibited all the other consortium members except for R. taiwanensis QL-117; R. mannitolilytica QL-A3 inhibited all other consortium members except for R. mannitolilytica QL-A2 and *R. taiwanensis* QL-117; and *R. taiwanensis* QL-117 inhibited all other consortium members except for *R. pickettii* QL-140 (Fig. 1B and Fig. S3). While the nonsiderophore metabolite-mediated facilitative interactions were more common (12 versus 7) (Fig. 1B and C, Fig. S3), interactions between consortium members were similar to the metabolite control treatment (only 6 out of 20 pairwise interactions showed slight differences) (Fig. 1A and C, Fig. S3). We also found that siderophore-mediated interactions between consortium members were similar to the interactions exerted by total metabolites (only 5 out of 20 pairwise interactions showed slight differences) (Fig. 1B and D, Fig. S3). Altogether, these results suggest that within-consortium interactions were less sensitive to siderophore production compared to consortia-pathogen interactions and that siderophores produced by *R. pickettii* QL-A6 may potentially act as public goods.

10.1128/mSystems.00811-19.3 FIG S3 Total metabolite (siderophore and nonsiderophore) effects of inoculated strains on each other’s growth. The direct total metabolite effect on the pathogen growth were measured under iron-rich (MC, purple) and iron-limited (SM, yellow) conditions. The nonsiderophore metabolite effects (M, cyan-blue) on the pathogen growth under iron-limited condition are expressed as the effect by iron-limited supernatant replenished with an excess of iron to chelate produced siderophores in iron-limited conditions. To quantify the effects of siderophores only (S, black), we subtracted the nonsiderophore metabolite effect from the total metabolite effect (SM − M = S). Panels A to E represent the effect of metabolites produced by QL-A2, QL-A3, QL-A6, QL-117, and QL-140 on each other, respectively. Data show the mean of four independent experiments and error bars indicate the standard deviation. Lowercase letters above each bar represent the significance of each strain’s effect under different treatments at *P* < 0.05 (Duncan’s multiple range test). Download FIG S3, TIF file, 1.7 MB. Copyright © 2020 Gu et al. 2020 Gu et al. This content is distributed under the terms of the Creative Commons Attribution 4.0 International license.

### Siderophore and nonsiderophore metabolite-mediated effects in pathogen-consortium communities.

**FIG 2:**
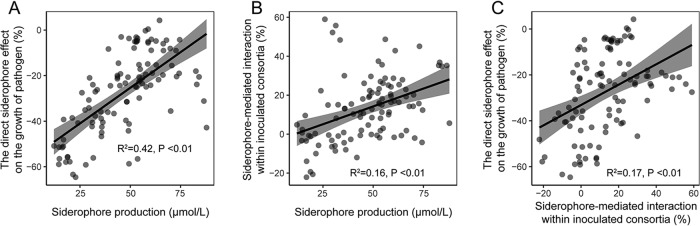
Siderophore-mediated effects on pathogen growth and interactions between consortium members. The direct siderophore effect on the pathogen growth (A) and siderophore-mediated interactions within inoculated consortia (B) correlate with siderophore production by the inoculated consortia. Direct siderophore-mediated effects on the pathogen growth correlate with siderophore-mediated interactions within inoculated consortia (C). In all panels, each point refers to a defined combination of consortium members.

At the whole-consortium level, we found that both the direct siderophore effect on pathogen growth (*R*^2^ = 0.42, *F_1_*_,102_ = 76.1, *P < *0.01) (Fig. 2A) and siderophore-mediated interactions within consortia (*R*^2^ = 0.16, *F*_1,102_ = 20.1, *P < *0.01) (Fig. 2B) positively correlated with the overall siderophore production. Siderophore-mediated interactions within consortia also positively correlated with siderophore-mediated suppression of the pathogen (*R*^2^ = 0.17, *F*_1,102_ = 21.7, *P* < 0.01) (Fig. 2C). However, there was a nonsignificant relationship between nonsiderophore metabolite-mediated interactions within inoculated consortia and between the nonsiderophore metabolite effects on the growth of the pathogen (*P* > 0.05) (Fig. S4). This suggests that nonsiderophore metabolite effects may be different for the whole consortium compared to single inoculant strains, as previously observed (Fig. 1C). We further explored the relative importance of community richness and strain identity effects on these relationships. Siderophore production of the inoculant strains increased with increasing consortium richness under iron-limited conditions (*R^2^* = 0.06, *F*_1,122_ = 9, *P* < 0.01, no relationship observed in iron-rich control conditions) (Table S1). However, strain richness only had a weak linear relationship with the siderophore-mediated pathogen suppression (*R*^2^ = 0.05, *F*_1,122_ = 7, *P* < 0.01) (Table S2) and a nonsignificant relationship with the siderophore-mediated interactions within inoculated consortia (not retained in the model) (Table S3). Instead, we found strong strain identity effects on siderophore production by the consortia. Specifically, the presence of strain *R. pickettii* QL-A6 had a significant positive effect (*P* < 0.001), strain *R. mannitolilytica* QL-A2 (*P* = 0.016) and *R. mannitolilytica* QL-A3 (*P* = 0.011) had significant negative effects, and strains *R. taiwanensis* QL-117 and *R. pickettii* QL-140 had nonsignificant neutral effects on siderophore production. In the case of pathogen suppression, we found that strains *R. pickettii* QL-A6 and *R. taiwanensis* QL-117 had positive effects and *R. mannitolilytica* QL-A2, *R. mannitolilytica* QL-A3, and *R. pickettii* QL-140 negative effects (all *P* < 0.05) (Table S2). These results suggest that siderophore-mediated effects were always relatively more important than nonsiderophore metabolite-mediated effects, and that strain identity effects were stronger compared to consortium richness effects in determining the strength of interactions within the consortium and between the pathogen and consortium.

10.1128/mSystems.00811-19.4 FIG S4 Relationship between nonsiderophore metabolite-mediated interactions within inoculated consortia and nonsiderophore metabolite effects on pathogen growth. There was no relationship between nonsiderophore metabolite-mediated interactions within inoculated consortia and the direct nonsiderophore metabolite effect on pathogen growth (*P* > 0.05). Download FIG S4, TIF file, 0.4 MB. Copyright © 2020 Gu et al. 2020 Gu et al. This content is distributed under the terms of the Creative Commons Attribution 4.0 International license.

10.1128/mSystems.00811-19.7 TABLE S1 General linear mixed model (GLM) analyzing the effects of the strain richness (*Model 1-diversity effects*) and strain identity (*Model 2-identity effects*) on the siderophore production by inoculated consortia under iron-limited and iron-rich conditions. In the identity effect model, the effect of each strain was analyzed as the presence versus absence of each strain within the consortia (as a binary predictor). All response variables were treated as continuous variables and the table shows the most parsimonious models selected based on the AIC information. The significant effects (*P* < 0.05) are highlighted in bold and the up and downwards arrows denote positive and negative effects on response variables, respectively. Download Table S1, DOCX file, 0.01 MB. Copyright © 2020 Gu et al. 2020 Gu et al. This content is distributed under the terms of the Creative Commons Attribution 4.0 International license.

10.1128/mSystems.00811-19.8 TABLE S2 General linear mixed model (GLM) analyzing the effects of the strain richness (*Model 1-diversity effects*) and strain identity (*Model 2-identity effects*) on the direct siderophore and nonsiderophore metabolite effects on the growth of pathogen. In the identity effect model, the effect of each strain was analyzed as the presence versus absence of each strain within the consortia (as a binary predictor). All response variables were treated as continuous variables and the table shows the most parsimonious models selected based on the AIC information. The significant effects (*P* < 0.05) are highlighted in bold and the up and downwards arrows denote positive and negative effects on response variables, respectively. Download Table S2, DOCX file, 0.01 MB. Copyright © 2020 Gu et al. 2020 Gu et al. This content is distributed under the terms of the Creative Commons Attribution 4.0 International license.

10.1128/mSystems.00811-19.9 TABLE S3 General linear mixed model (GLM) analyzing the effects of the strain richness (*Model 1-diversity effects*) and strain identity (*Model 2-identity effects*) on the siderophore and nonsiderophore metabolite-mediated interactions within inoculated consortia. In the identity effect model, the effect of each strain was analyzed as the presence versus absence of each strain within the consortia (as a binary predictor). All response variables were treated as continuous variables and the table shows the most parsimonious models selected based on the AIC information. The significant effects (*P* < 0.05) are highlighted in bold and the up and downwards arrows denote positive and negative effects on response variables, respectively. Download Table S3, DOCX file, 0.01 MB. Copyright © 2020 Gu et al. 2020 Gu et al. This content is distributed under the terms of the Creative Commons Attribution 4.0 International license.

### Using siderophore and nonsiderophore metabolite-mediated interactions to predict disease incidence in the tomato rhizosphere.

**FIG 3:**
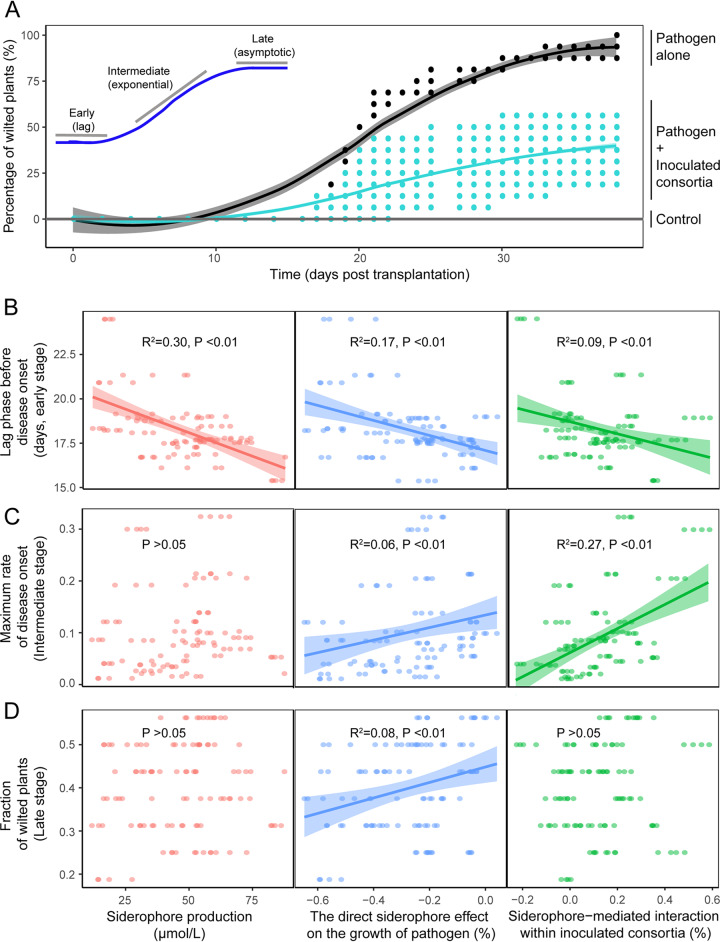
The suppressiveness of inoculated consortia for the disease incidence progression in tomato plant rhizosphere. (A) Progression of bacterial wilt plant disease in the absence (black line) and presence of inoculated consortia (teal line). Disease spread was fitted with the data using a logistic regression to obtain three variables describing the dynamics of disease: lag time before disease onset (early stage), the maximum rate of disease onset (intermediate stage), and fraction of wilted plants (late stage). (B to D) Siderophore production (left), the direct siderophore effect on the growth of pathogen (middle), and siderophore-mediated interactions within inoculated consortia (right) on the disease progression during different stages of infection. In all panels, each point refers to a defined combination of consortia members.

The proportion of wilted plants increased with time during the greenhouse experiment and the disease spread fitted well with a logistic regression (Fig. 3A). While the presence of inoculated consortia significantly decreased the disease incidence, this effect depended on its composition (58% reduction, *F*_1,125_ = 82, *P* < 0.01) (Fig. 3A). While siderophore production, siderophore effects on pathogen growth, and siderophore-mediated interactions within inoculated consortia all significantly explained the disease spread, the relative importance of each factor varied along with the different stages of infection (the effect of consortia strain richness was nonsignificant, *P > *0.05) (Fig. 3B to D and Table 1, Table S4). During the early stage of infection before the disease onset (lag phase), siderophore production by inoculated consortia was the main factor predicting the consortium suppressiveness (*R*^2^ = 0.30, *P* < 0.01) (Fig. 3B and Table 1). In contrast, during the intermediate disease stage, the siderophore-mediated interactions within inoculated consortia were the most important factor influencing the maximum rate of disease onset (*R*^2^ = 0.27) (Fig. 3C and Table 1). During the last disease stage, only the siderophore effect on the pathogen growth significantly explained the proportion of wilted plants (*R*^2^ = 0.08) (Fig. 3D and Table 1). Moreover, the effect of siderophore production became nonsignificant during the intermediate and last disease stages, while siderophore-mediated interactions within inoculated consortia became nonsignificant during the last disease stage. The nonsiderophore metabolite-mediated effects on pathogen growth and nonsiderophore metabolite-mediated interactions within inoculated consortia had nonsignificant effects on disease incidence (*P* > 0.05) (Fig. S5). Furthermore, we found that the strain identity had a strong effect on the lag phase before the disease onset (*R*^2^=0.31 and *P* < 0.01) (Table S4). Specifically, the presence of strains *R. pickettii* QL-A6 and *R. taiwanensis* QL-117 reduced the lag time, strains *R. mannitolilytica* QL-A3 and *R. pickettii* QL-140 increased the lag time, and the strain *R. mannitolilytica* QL-A2 had a nonsignificant effect (Table S4). Together, these results suggest that siderophore-mediated effects on disease incidence were stronger compared to nonsiderophore metabolite-mediated effects, while these effects varied depending on the stage of infection and the presence of certain species in the inoculated consortium.

**TABLE 1:** General linear mixed model (GLM) comparing contributions of siderophore production, the direct siderophore effect and siderophore-mediated interactions on the dynamics of disease progression *in planta*^*a*^

Variable	Lag phase before disease onset			Maximum rate of disease onset			Fraction of wilted plants		
	df	*F*	*P*	df	*F*	*P*	df	*F*	*P*
Siderophore production	**↓1**	**44.43**	**<0.01**	1	2.57	0.2	1	1.72	0.19
Direct siderophore effect	1	1.64	0.2	1	1.64	0.11	**↑1**	**9.99**	**<0.01**
Siderophore-mediated interaction	1	0.47	0.49	**↑1**	**39.27**	**<0.01**	1	0.01	0.93
No. of residuals		100			100			100	
Model summary	*R*^2^: 0.30; AIC: 384.33			*R*^2^: 0.28; AIC: −253.80			*R*^2^: 0.08; AIC: −174.09		

a All response variables were treated as continuous variables. The table shows the most parsimonious models selected based on the Akaike information criterion (AIC) information. The upward and downward arrows denote positive and negative effects on response variables, respectively and the significant effects (*P* < 0.05) are highlighted in bold.

10.1128/mSystems.00811-19.5 FIG S5 Determining the suppressiveness of inoculated consortia in the tomato rhizosphere. (A to C) The direct nonsiderophore metabolite effect on the growth of pathogen and the disease development during each stage of infection. (D to F) The effect of nonsiderophore metabolite-mediated interaction within inoculated consortia on the disease development during each stage of infection. Download FIG S5, TIF file, 1.9 MB. Copyright © 2020 Gu et al. 2020 Gu et al. This content is distributed under the terms of the Creative Commons Attribution 4.0 International license.

10.1128/mSystems.00811-19.10 TABLE S4 General linear mixed model (GLM) analyzing the effects of the strain richness (*Model 1-diversity effects*) and strain identity (*Model 2-identity effects*) on the dynamics of disease. In the identity effect model, the effect of each strain was analyzed as the presence versus absence of each strain within the consortia (as a binary predictor). All response variables were treated as continuous variables and the table shows the most parsimonious models selected based on the AIC information. The significant effects (*P* < 0.05) are highlighted in bold and the up and downwards arrows denote positive and negative effects on response variables, respectively. Download Table S4, DOCX file, 0.01 MB. Copyright © 2020 Gu et al. 2020 Gu et al. This content is distributed under the terms of the Creative Commons Attribution 4.0 International license.

## DISCUSSION

Synthetic microbial inoculants have been proposed as a way to suppress pathogens and enhance plant health (37, 38). However, the outcomes of such manipulations still vary considerably due to poor establishment of the inoculants, which could be caused by mismatches in the ability to grow and survive in local abiotic and biotic environmental conditions in the rhizosphere (27, 28). As iron is often an important limiting resource in soil, we hypothesized that bacterial interactions mediated by iron-scavenging siderophores could be used to predict the disease suppressiveness and success of microbial inoculants. We found that siderophores produced by the inoculated consortia played a more important role in pathogen growth compared to other secondary, nonsiderophore metabolites, indicative of their importance for pathogen suppression. Furthermore, while siderophore-mediated interactions and inoculant strain identity effects played an important role in explaining pathogen invasion in the plant rhizosphere, the effects of consortium richness had no effect. As a result, depending on the consortium composition, siderophore-mediated effects could either facilitate or suppress the pathogen growth, likely depending on the specificity of siderophores. Our findings hence suggest that siderophore-mediated interactions within inoculated consortia and between the consortium and the pathogen are important in predicting the effect of microbial inoculants on pathogen suppression.

Previous studies have shown that both siderophores and antibiotics are important for suppressing the growth of pathogens (39, 40). Our results show that siderophores play a much more important role in mediating effects on *Ralstonia solanecearum* growth compared to nonsiderophore metabolites, and no direct inhibition typical for antibiosis was observed. One potential explanation for these results is that all the strains included in the consortia belonged to the genus *Ralstonia* and, due to this high relatedness, produced and were resistant to similar antimicrobials (13). Moreover, as iron is essential for microbial metabolism, numerous secondary metabolites, such as antibiotics, may not be expressed at high levels under limiting iron conditions (41), increasing the relative importance of siderophores under such conditions. Furthermore, we have previously found that resource competition is the main factor in mediating effects between the same consortium members and the pathogen for disease suppression (13, 28, 42). This study thus suggests that interactions between these bacteria could be driven by a combination of iron and carbon competition.

Recent studies have shown that siderophores can mediate both antagonistic and facilitative effects in microbial communities depending on whether other microbes have the matching receptors to take up heterologous siderophores (43–47). In this study, we used a relative comparison of siderophore effects using a well-established chrome azurol S (CAS) assay that indirectly measures the siderophore effects without quantifying the absolute number or type of siderophores. Our results suggest that inoculated strains can either inhibit or promote the pathogen, and the resulting disease outbreaks potentially depend on the compatibility of siderophores produced by each strain. Other studies have reported that incompatible siderophores could potentially constrain pathogen infection by reducing available iron in the environment (48–51). Although bacteria belonging to the same genus may share siderophores as public goods, e.g., *Pseudomonas* (45), strain-specific siderophores may be produced to avoid intraspecific competition and exploitation by social cheats (52, 53). As shown here, despite that the consortium members and the pathogen all belonged to the genus *Ralstonia*, all but one strain, *R. pickettii* QL-A6, produced siderophores that had antagonistic effects on the growth of the pathogen. These results thus support the idea that siderophores produced by closely related bacterial strains that potentially share the same ecological niche are likely to be private goods and accessed only by themselves. While the direct purification of siderophores and deciphering of their structural diversity remains challenging (54), in the future it will be important to characterize siderophore-mediated effects observed in this study at the molecular and genome level (50). In addition to simplified lab studies, siderophore production should be quantified *in vivo* in complex rhizosphere bacterial communities and the survival of inoculated strains determined using strain-specific markers (55), allowing better understanding of siderophore-mediated competition in field conditions.

As previously demonstrated, interactions within inoculated consortia can reliably predict the likelihood of microbial invasions (12). However, the exact mechanisms driving these interactions between the inoculated consortium and the pathogen often remain unclear. While siderophore-mediated bacterial interactions have been shown to be important in natural environments, and within eukaryotic hosts under iron-limiting conditions (45, 56), our findings provide an ecological explanation of how siderophore-mediated bacterial interactions could predict plant disease dynamics in the agricultural context. Specifically, even though most of the pairwise interactions between the consortium members and the pathogen were suppressive, the especially strong facilitative effect by the strain QL-A6 on the pathogen could overshadow the suppressive effects of otherwise suppressive consortia. This result emphasizes the importance of strain identity when choosing strains for microbial inoculants. It is also worth noting that the explanatory power of siderophore-mediated interactions in the rhizosphere was moderate. Considering the complexity of natural systems, and lack of control of several confounding and highly variable factors, this finding demonstrates that siderophore-mediated interactions are likely to be very important for governing microbial interactions in the soil. The predictive power of these analyses could potentially be improved via incorporation of multiple interactions, such as different forms of resource competition, siderophore production *in vivo*, and inoculant survival success to achieve a more thorough and robust predictive framework for pathogen invasions in complex rhizosphere environments (13, 24).

In conclusion, our results suggest that iron-scavenging siderophores can both promote or constrain pathogen invasion depending on the consortium composition, which ultimately determines the net strength and direction of siderophore-mediated interactions. Hence, direct effects of siderophores on pathogen growth and the siderophore-mediated interactions within inoculated consortia are both important for predicting pathogen suppression. We hope these results can be broadly applied to control a wide range of microbial infections, both soilborne and human-related, as iron exploitation is an important factor for pathogen colonization and infection with many hosts (57–59). In the context of functional microbial inoculant design, we suggest that including strains that trigger strong siderophore-mediated competitive interactions into inoculant consortia is important for reaching high biocontrol efficacy. For example, consortia whose siderophores can be used for the growth of nonpathogenic species, but which are inaccessible to the pathogen, might allow potentially stable coexistence of nonpathogenic competitors and strong continuous suppression of the pathogen.

## MATERIALS AND METHODS

### Bacterial strains and construction of inoculated consortia.

We used Ralstonia solanacearum strain QL-Rs1115 (GenBank accession GU390462) tagged with the pYC12-mCherry plasmid (13) as a model pathogen in our study. The inoculated consortia comprised of five closely related *Ralstonia* strains (Ralstonia mannitolilytica QL-A2, Ralstonia mannitolilytica QL-A3, Ralstonia pickettii QL-A6, *Ralstonia taiwanensis* QL-117, and Ralstonia pickettii QL-140) that have previously been shown to inhibit pathogen growth solely via resource competition without detectable toxin production (13). For the experiments, one colony of each strain, recovered from –80°C 20% glycerol stocks, was selected and grown in nutrient broth (NB, glucose 10.0 g liter^−1^, tryptone 5.0 g liter^−1^, beef extract 3.0 g liter^−1^, yeast extract 0.5 g liter^−1^, pH 7.0) with 170-rpm agitation at 30°C for 12 h. Bacteria were then washed three times by centrifugation (5,000 rpm, 5 min), resuspended in 0.85 % NaCl, and adjusted to a density of 10^7^ cells ml^−1^. Inoculated consortia were constructed by using a full factorial design, including all possible strain combinations at one to five strain richness levels (for a total of 31 communities) following a substitutive design where all consortia had the same final total bacterial densities (10^7^ cells ml^−1^) and equal ratios of all included strains (12).

### Determining the siderophore production of the inoculated consortia and consortia members.

The chrome azurol S (CAS) assays (60) were used to measure siderophore production in iron-rich and iron-limited conditions. Even though the CAS assays does not measure the type and absolute amount of siderophores produced, it is a well-established method for relative comparisons between different strains and communities (61). To establish a control baseline for no siderophore production in the CAS assay, we used two siderophore-negative mutants (Pseudomonas aeruginosa and Burkholderia cenocepacia strains). These siderophore-negative strains showed a mean siderophore production of 6.67 and 7.67 μM under iron-limited and iron-rich conditions, respectively (61). These values were thus considered the background signal of no siderophore production in CAS assays in both conditions. Siderophore production of inoculated consortia and defined siderophore-negative mutants of Pseudomonas aeruginosa and Burkholderia cenocepacia strains were tested in MKB medium (K_2_HPO_4_ 2.5 g liter^−1^, MgSO_4_·7H_2_O 2.5 g liter^−1^, glycerinum 15 ml liter^−1^, casamino acids 5.0 g liter^−1^, pH 7.2) under both iron-limited and iron-rich conditions. The iron-rich condition was achieved by adding iron (III) solution (1 mM FeCl_3_·6H_2_O, 10 mM HCl) into MKB medium (final concentration equaling 50 μM). Each inoculated strain or consortium was grown in both iron-limited and iron-rich MKB medium using 96-well microplate assays. The wells contained a total of 200 μl of liquid consisting of 185 μl of MKB medium and 15 μl of inoculum of each constructed consortium (10^7^ cells ml^−1^) and were incubated at 30°C with 170-rpm orbital agitation for 48 h, which allowed all consortia to reach stationary phase. The cell-free supernatant was obtained by centrifugation (10,000 rpm, 5 min) and filtration (0.22-micron filter) and siderophore production was measured using a universal CAS chemical assay. Briefly, 100 μl of each cell-free supernatant, or fresh medium as a control, were added to 100 μl of CAS assay solution in a 96-well microplate. After 2 h of static cultivation at room temperature, the optical density at 630 nm (OD_630_) of cell-free supernatants and uninoculated medium controls were measured using a spectrophotometer (SpectraMax M5, Sunnyvale, CA, USA). Siderophore production was estimated using a reference curve based on the relationship between OD_630_ values and known desferoxamine siderophore (EmergenBio) concentrations increasing from 0 to 100 μg ml^−1^.

### Testing siderophore effects on pathogen growth and interactions between consortium members.

**(i) Measuring siderophore and nonsiderophore metabolite effects on the pathogen growth.** The R. solanacearum pathogen strain QL-Rs1115 was exposed to supernatants produced by all strains and consortia to test the effects of siderophores and other secondary metabolites to its growth (Fig. S6). To disentangle these effects, we used three types of supernatant treatments. (i) Strain and consortia were grown in iron-limited MKB medium to trigger siderophore production. This supernatant contained both siderophore and nonsiderophore metabolites and is henceforth referred as siderophore-metabolite supernatant (SM). (ii) To test the effect of nonsiderophore metabolites on pathogen growth, we treated SM supernatant with 50 μM FeCl_3_ to chelate siderophores via an iron-chelation reaction. This supernatant contained only nonsiderophore secondary metabolites and is henceforth referred to as nonsiderophore metabolite supernatant (M). (iii) Finally, we also used a metabolite-control supernatant (MC) where we grew individual strains and consortia in iron-rich conditions to obtain supernatant with secondary metabolites but very few siderophores. This control was used as a positive control for the iron-chelation treatment. In addition, sterilized water was used as a control instead of the supernatant (C).

10.1128/mSystems.00811-19.6 FIG S6 Overview of the experimental design used to assess the effect of siderophores produced by strains and consortia on the growth of Ralstonia solanacearum. To explore how competition from iron affected interactions between the pathogen and consortia, we grew the pathogen in triplicates in the presence of supernatant from each of our consortia. To determine whether the supernatant-mediated effects on pathogen growth were due to iron competition or to nonsiderophore metabolites, we set up three different types of supernatant treatments: (i) Strain and consortia were grown in iron-limited MKB medium to trigger siderophore production. This supernatant contained both siderophores and nonsiderophore metabolites and is henceforth referred to as siderophore-metabolite supernatant (SM). (ii) To test the effect of nonsiderophore metabolites on pathogen growth, we treated SM supernatant with 50 μM FeCl_3_ to chelate produced siderophores via an iron-chelation reaction. This supernatant contained nonsiderophore metabolites and is henceforth referred to as nonsiderophore metabolite supernatant (M). (iii) We also used a metabolite-control supernatant (MC) in which we grew strain and consortia in iron-rich conditions to obtain supernatant with secondary metabolites but only very few siderophores. This control was used as a positive control for the iron-chelation treatment. In addition, sterilized water was used as a control instead of the supernatant (C). We then measured the effect of each supernatant on pathogen growth and calculated the net effect of siderophores by subtracting the growth effect of the iron-replenished supernatant (SM) from the growth effect of the iron-limited supernatant (M). The effect of each supernatant on pathogen growth (SNG) was calculated as a relative growth effects (RG) compared to the water control (CG) treatment as follows: RG=([SNG/CG]-1) × 100, where SNG refers to SM, M, and MC. Values smaller and greater than zero indicate growth inhibition and facilitation, respectively, expressed as percentage fold-change. Download FIG S6, TIF file, 1.7 MB. Copyright © 2020 Gu et al. 2020 Gu et al. This content is distributed under the terms of the Creative Commons Attribution 4.0 International license.

To test the effect of siderophores and nonsiderophore metabolites on pathogen growth in iron-limited conditions, we grew the pathogen in iron-limited medium with supernatant that was derived from iron-limited conditions (i; SM). To test the effect of nonsiderophore metabolites in iron-limited conditions, we grew the pathogen in iron-rich medium with supernatant that was derived from iron-limited conditions and “inactivated” with FeCl_3_ (ii; M). To test the effect of nonsiderophore metabolites in iron-rich conditions, we grew the pathogen in iron-rich medium with supernatant that was derived from iron-rich conditions (iii; MC). All measurements were conducted in 96-well microplates with 180 μl of 10% MKB medium, 20 μl of cell-free consortia supernatants, and 2 μl of inoculant of overnight pathogen culture (adjusted to OD_600_ = 0.5 after 12 h growth at 30°C with shaking). Pathogen-supernatant cultures were incubated with shaking (rotary shaker set at 170 rpm min^−1^) at 30°C and the change in pathogen density was measured as optical density with a spectrophotometer after 24 h of growth (OD_600_) (SpectraMax M5 plate reader). The effect of each supernatant on the pathogen growth (SNG) was calculated as the relative growth effect (RG) compared to the water-control (CG) treatment as follows: RG = ([SNG ÷ CG] − 1) × 100, where SNG refers to SM, M, and MC supernatants as described above. RG values below or above zero thus indicated growth inhibition and facilitation, respectively, and were expressed as percentage fold changes. To quantify the effects of siderophores only (S), we used an index based on the two supernatant treatments above by subtracting the metabolite effect from the nonsiderophore metabolite effects (SM − M = S).

**(ii) Determining siderophore and nonsiderophore metabolite-mediated pairwise interactions between inoculated consortium members.** To quantify the strength and direction of each pairwise interaction within consortia, we tested how the supernatant of each member affected the growth of the other four consortium members. Briefly, we used the same experimental setup to obtain S, M, and SM supernatant as described above to disentangle siderophore and nonsiderophore metabolite effects from each other. For each of the five consortium members, 2 μl of overnight cultures (adjusted to OD_600_ = 0.5 after 12 h of growth at 30°C with shaking) were separately added into microplate wells containing 180 μl of 10% MKB medium and 20 μl of cell-free supernatant of the other strains (all pairwise interactions were measured individually). The effect of different supernatants on the growth of consortium members was calculated as the relative growth effect compared to the water-control treatment, as described above.

**(iii) Determining siderophore and nonsiderophore metabolite-mediated growth effects by the multistrain consortia on individual consortium members.** To calculate the mean siderophore and nonsiderophore metabolite-mediated growth effect by multistrain consortia on each consortium member, we grew all inoculated strains individually in the presence of supernatant mix produced by all other strains present in every given consortium. For example, in consortium A+B+C, we measured the consortium supernatant effects individually against A, B, and C using the same methodology as described previously. These pairwise consortia-consortia member interactions were used to calculate the mean intensity of siderophore-mediated interactions within the consortium as an average of these pairwise interactions.

### Determining the effects of inoculated consortia on bacterial wilt disease incidence in tomato rhizosphere.

To quantify the suppressiveness of all inoculated consortia on the pathogen *in vivo*, we used a 49-day greenhouse experiment with tomato to measure changes in bacterial wilt disease progression in the presence of 31 inoculated consortia (including a positive control containing only the pathogen and a negative control without any bacteria; all treatments were replicated three times). Surface-sterilized tomato seeds (Lycopersicon esculentum, cultivar ‘Jiangshu’) were germinated on water-agar plates for 3 days before sowing into seedling plates containing seedling substrate (Huainong, Huaian soil and Fertilizer Institute, Huaian, China). Tomato plants were transplanted to seedling trays containing the natural soil from a rice field in Wuxi (Jiangsu Province, China) at the three-leaf stage (11 days after sowing) and inoculated with bacterial consortia using the drenching method (final concentration of 10^8^ cells per g of soil). The pathogen was inoculated to the rhizosphere 1 week later using the drenching method (final concentration of 10^6^ CFU per g soil) and tomato plants were grown for 38 days in a greenhouse with natural temperature variation ranging from 25 to 35°C and watered regularly. Seedling plates were arranged in a randomized order and rearranged randomly every 2 days. The number of wilted plants per seedling plate was used to determine disease severity as a disease index on a daily basis beginning 17 days post transplantation and after the first symptoms became visible.

### Statistical analyses.

General linear mixed models were used to examine the siderophore and nonsiderophore metabolite effects on the pathogen and consortium member growth in mono, pairwise, and consortia cocultures. In these analyses, we conducted separate models to explore consortium community richness, composition and strain identity effects (i.e., the presence and/or absence of certain strains) on strain interactions. Differences in disease incidence were explained by three quantitative factors that were measured *in vitro*: consortia siderophore production, consortia siderophore-mediated effect on the pathogen growth, and siderophore-mediated interactions between inoculated consortium members. All variables were fitted as continuous variables and one separate model was used for each variable. All analyses were performed using R 3.3.1 program (www.r-project.org).

### Data availability.

All data have been deposited to Dryad digital repository with the following digital identifier: https://doi.org/10.5061/dryad.6djh9w0xw.
