# Supplementary figures and images for: Siderophore-Mediated Interactions Determine the Disease Suppressiveness of Microbial Consortia
Source: mSystems. 2020 Jun 30;5(3):e00811-19. doi: 10.1128/mSystems.00811-19 (PMC7329327; doi:10.1128/mSystems.00811-19)

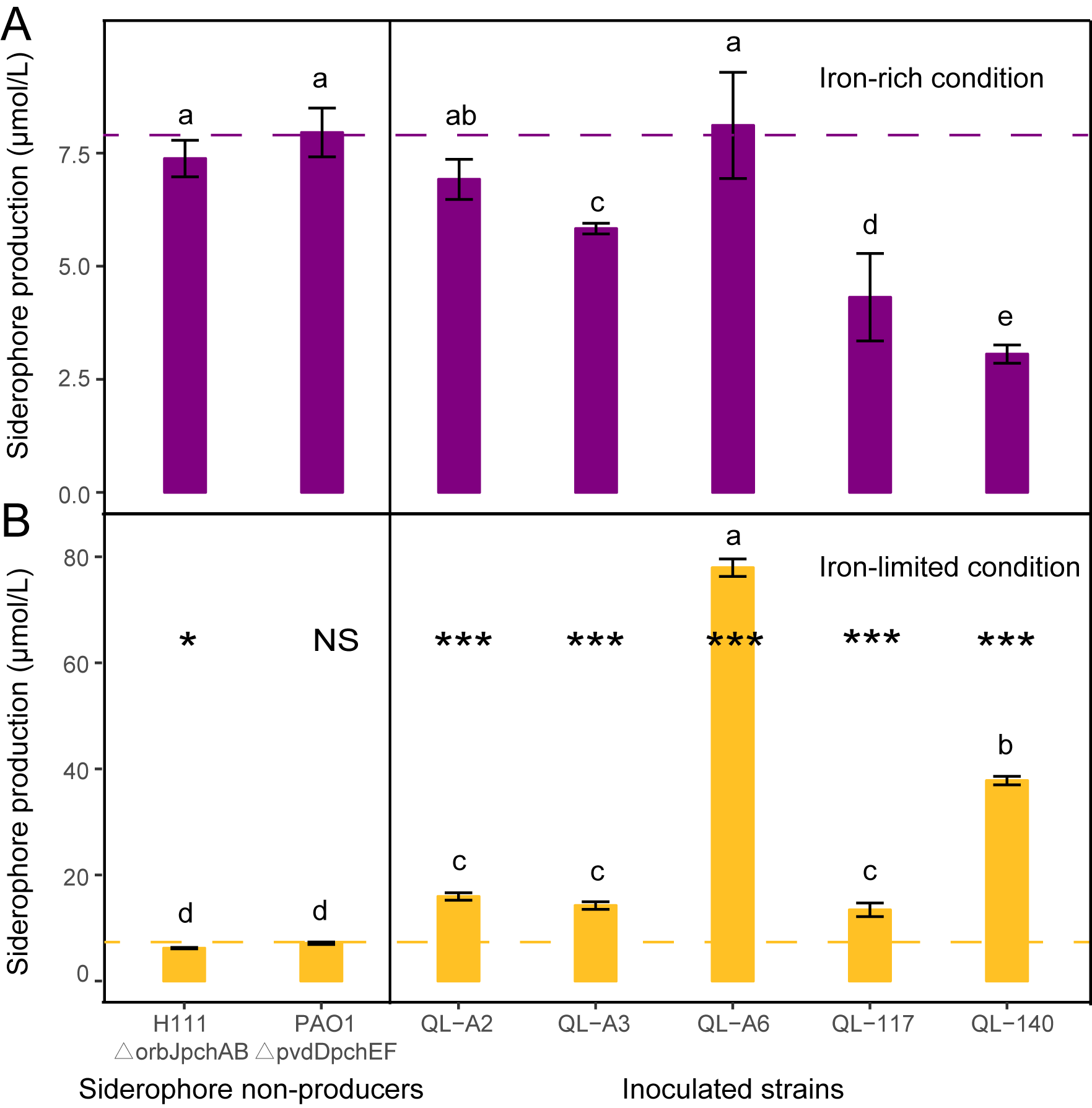

Supplement: FIG S1 [file mSystems.00811-19-sf001.tif]

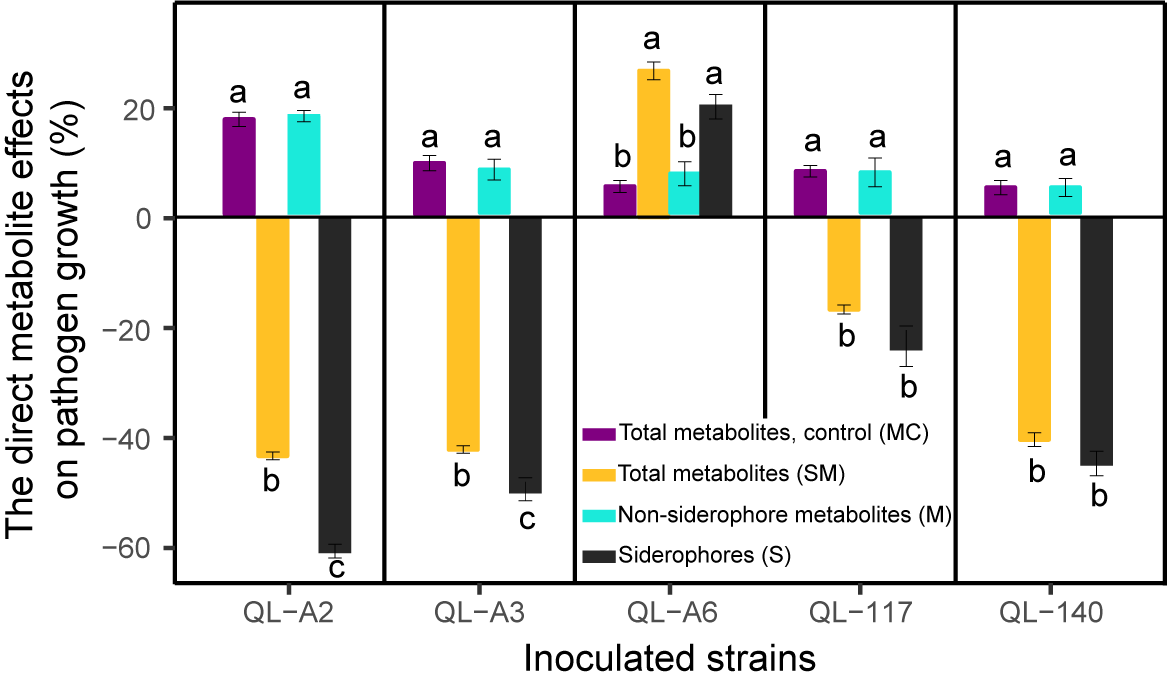

Supplement: FIG S2 [file mSystems.00811-19-sf002.tif]

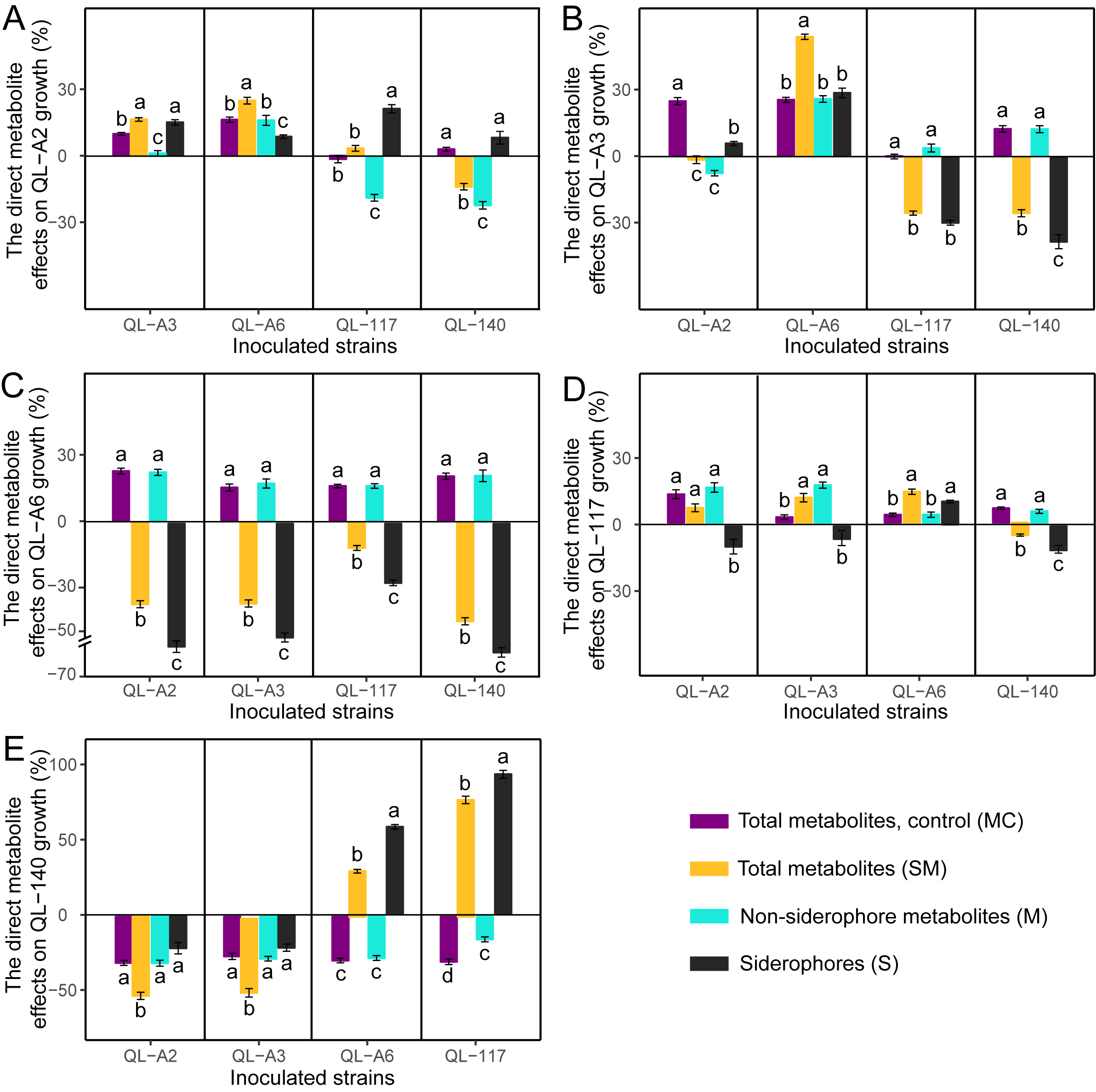

Supplement: FIG S3 [file mSystems.00811-19-sf003.tif]

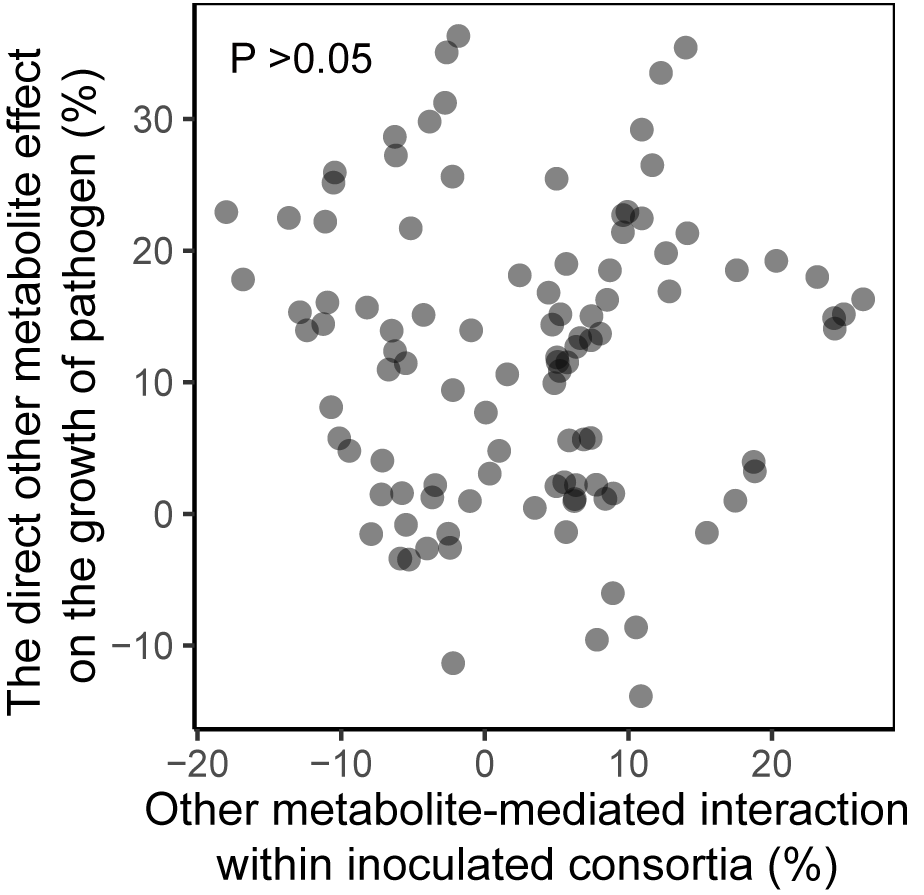

Supplement: FIG S4 [file mSystems.00811-19-sf004.tif]

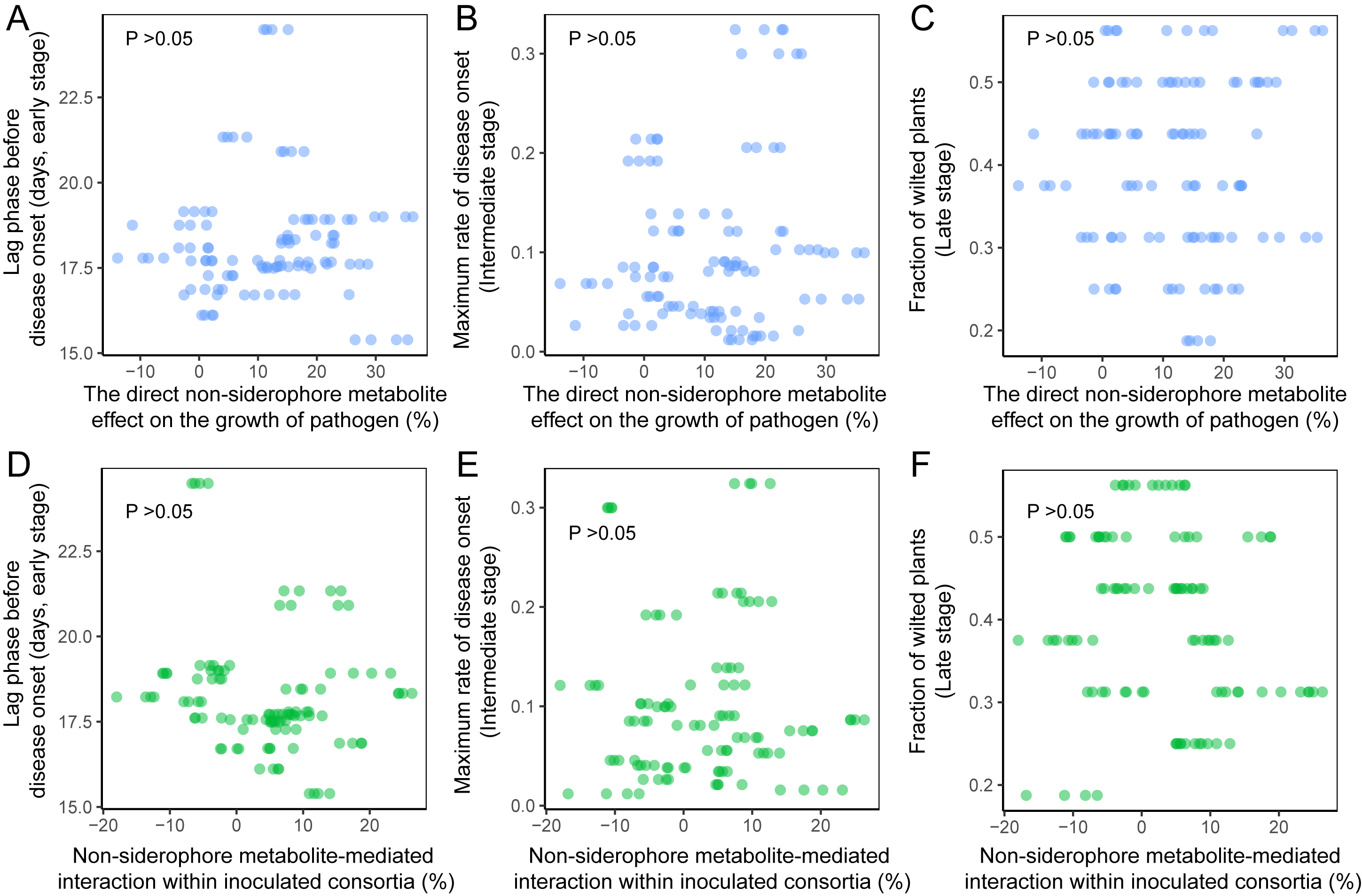

Supplement: FIG S5 [file mSystems.00811-19-sf005.tif]

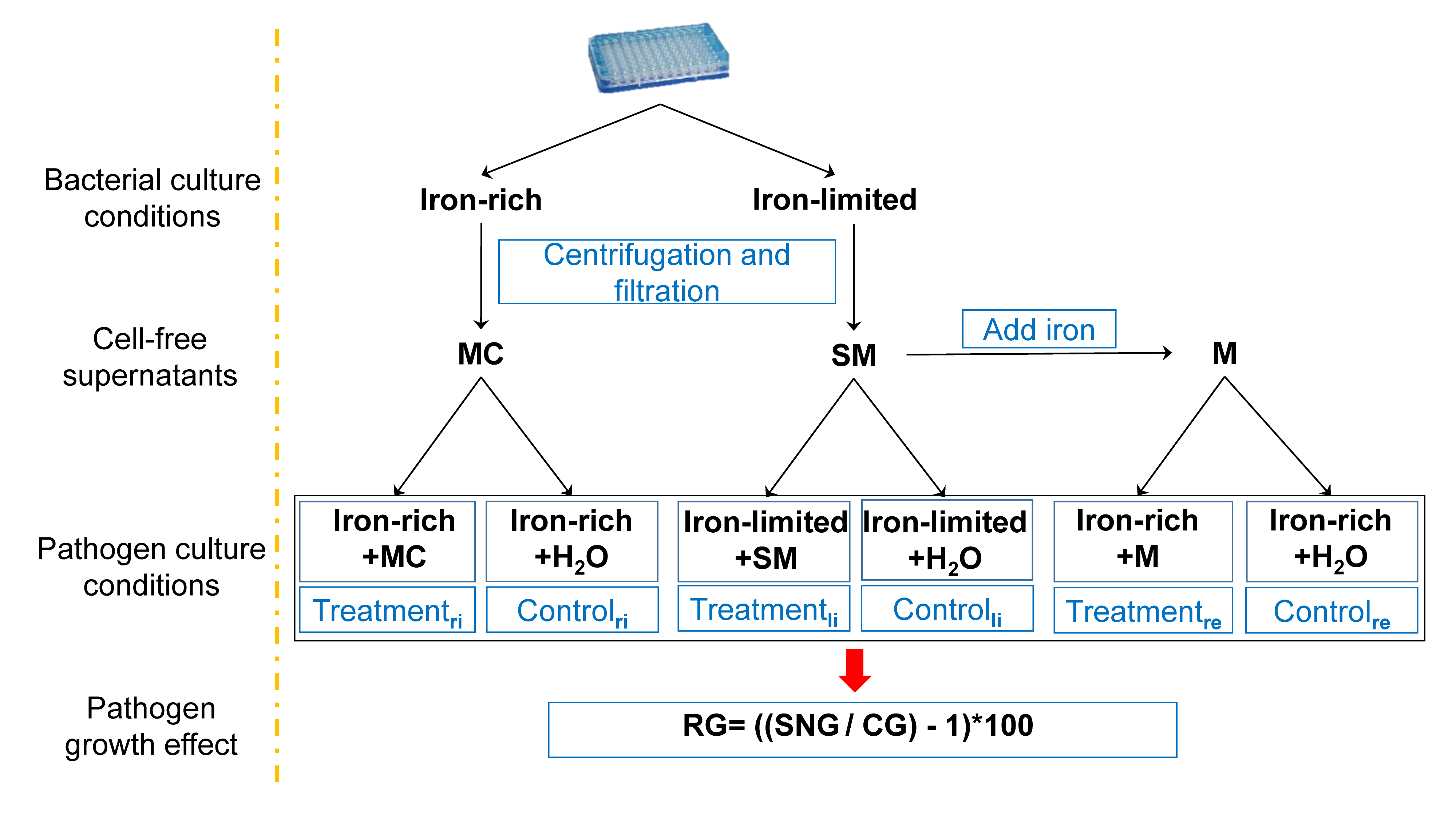

Supplement: FIG S6 [file mSystems.00811-19-sf006.tif]
