# Supplementary material for: Siderophore-Mediated Interactions Determine the Disease Suppressiveness of Microbial Consortia
Source: mSystems. 2020 Jun 30;5(3):e00811-19. doi: 10.1128/mSystems.00811-19 (PMC7329327; doi:10.1128/mSystems.00811-19)
Supplement: TABLE S1 [file mSystems.00811-19-st001.docx]

|  | Siderophore production under iron-limited condition | | | Siderophore production under iron-rich condition | | |
| --- | --- | --- | --- | --- | --- | --- |
|  | df | F | P | df | F | P |
| ***Model 1-diversity effects*** |  |  |  |  |  |  |
| Strain richness | **↑1** | **9** | **0.004** |  | Not retained |  |
| No. of Residuals |  | 122 |  |  |  |  |
| Model summary | R^2^:0.06 AIC:1668 | | |  | | |
| ***Model 2-identity effects*** |  |  |  |  |  |  |
| QL-A2 | **↓1** | **9** | **0.003** |  | Not retained |  |
| QL-A3 | **↓1** | **15** | **<0.001** |  | Not retained |  |
| QL-A6 | **↑1** | **207** | **<0.001** | **↑1** | **44** | **<0.001** |
| QL-117 |  | Not retained |  | **↓1** | **27** | **<0.001** |
| QL-140 | 1 | 2 | 0.1182 |  | Not retained |  |
| No. of Residuals |  | 119 |  |  | 119 |  |
| Model summary | R^2^:0.65 AIC:1547 | | | R^2^:0.35 AIC:963 | | |
