# Supplementary material for: Siderophore-Mediated Interactions Determine the Disease Suppressiveness of Microbial Consortia
Source: mSystems. 2020 Jun 30;5(3):e00811-19. doi: 10.1128/mSystems.00811-19 (PMC7329327; doi:10.1128/mSystems.00811-19)
Supplement: TABLE S2 [file mSystems.00811-19-st002.docx]

|  | The direct siderophore effect on the growth of pathogen | | | The non-siderophore metabolite effects on the growth of pathogen | | |
| --- | --- | --- | --- | --- | --- | --- |
|  | df | F | P | df | F | P |
| ***Model 1-diversity effects*** |  |  |  |  |  |  |
| Strain richness | **↑1** | **7** | **0.008** |  | Not retained |  |
| No. of Residuals |  | 122 |  |  |  |  |
| Model summary | R^2^:0.05 AIC:-50 | | |  | | |
| ***Model 2-identity effects*** |  |  |  |  |  |  |
| QL-A2 | **↓1** | **20** | **<0.001** | **↑1** | **6** | **0.017** |
| QL-A3 | **↓1** | **17** | **<0.001** | **↓1** | **4** | **0.04** |
| QL-A6 | **↑1** | **295** | **<0.001** | **↑1** | **6** | **0.017** |
| QL-117 | **↑1** | **30** | **<0.001** | **↓1** | **82** | **<0.001** |
| QL-140 | **↓1** | **7** | **0.007** | **↑1** | **27** | **<0.001** |
| No. of Residuals |  | 118 |  |  | 118 |  |
| Model summary | R^2^:0.65 AIC:-211 | | | R^2^:0.49 AIC:-286 | | |
