# Supplementary material for: Siderophore-Mediated Interactions Determine the Disease Suppressiveness of Microbial Consortia
Source: mSystems. 2020 Jun 30;5(3):e00811-19. doi: 10.1128/mSystems.00811-19 (PMC7329327; doi:10.1128/mSystems.00811-19)
Supplement: TABLE S3 [file mSystems.00811-19-st003.docx]

|  | Siderophore-mediated interaction within consortia | | | Non-siderophore metabolite-mediated interactions within consortia | | |
| --- | --- | --- | --- | --- | --- | --- |
|  | df | F | P | df | F | P |
| ***Model 1-diversity effects*** |  |  |  |  |  |  |
| Strain richness |  | Not retained |  |  | Not retained |  |
| No. of Residuals |  |  |  |  |  |  |
| Model summary |  | | |  | | |
| ***Model 2-identity effects*** |  |  |  |  |  |  |
| QL-A2 | **↓1** | **6** | **0.016** |  | Not retained |  |
| QL-A3 | **↓1** | **7** | **0.011** |  | Not retained |  |
| QL-A6 | **↑1** | **12** | **<0.001** | **↑1** | **52** | **<0.001** |
| QL-117 |  | Not retained |  |  | Not retained |  |
| QL-140 | **↑1** | **6** | **0.019** | **↓1** | **38** | **<0.001** |
| No. of Residuals |  | 99 |  |  | 101 |  |
| Model summary | R^2^:0.20 AIC:-94 | | | R^2^:0.46 AIC:-246 | | |
