# Supplementary material for: Siderophore-Mediated Interactions Determine the Disease Suppressiveness of Microbial Consortia
Source: mSystems. 2020 Jun 30;5(3):e00811-19. doi: 10.1128/mSystems.00811-19 (PMC7329327; doi:10.1128/mSystems.00811-19)
Supplement: TABLE S4 [file mSystems.00811-19-st004.docx]

|  | Lag phase before disease onset | | | Maximum rate of disease onset | | | Fraction of  wilted plants | | | |
| --- | --- | --- | --- | --- | --- | --- | --- | --- | --- | --- |
|  | df | F | P | df | F | P | df | F | P |  |
| ***Model 1-diversity effects*** |  |  |  |  |  |  |  |  |  |  |
| Strain richness | 1 | 0.25 | 0.62 | 1 | 0.84 | 0.36 | 1 | 3.49 | 0.06 |  |
| No. of Residuals |  | 118 |  |  | 118 |  |  | 118 |  |  |
| Model summary | R^2^:-0.006 AIC:473.67 | | | R^2^:-0.001 AIC:-268 | | | R^2^:0.019 AIC:-207 | | | |
| ***Model 2-identity effects*** |  |  |  |  |  |  |  |  |  |  |
| QL-A2 | 1 | 0.01 | 0.93 | 1 | 0.21 | 0.65 | 1 | 1.18 | 0.23 |  |
| QL-A3 | **↑1** | **11.93** | **<0.01** | 1 | 4.08 | 0.05 | **↓1** | **6.32** | **0.02** |  |
| QL-A6 | **↓1** | **33.62** | **<0.01** | 1 | 0.79 | 0.38 | 1 | 2.22 | 0.10 |  |
| QL-117 | **↓1** | **6.17** | **0.014** | 1 | 0.07 | 0.79 | **↑1** | **6.02** | **0.01** |  |
| QL-140 | **↑1** | **6.55** | **0.012** | 1 | 0.23 | 0.64 | 1 | 2.68 | 0.10 |  |
| No. of Residuals |  | 114 |  |  | 114 |  |  | 114 |  |  |
| Model summary | R^2^:0.31 AIC:432.37 | | | R^2^:0.003 AIC:-264.59 | | | R^2^:0.098 AIC:-214 | | | |
